# Impact of climate change on biodiversity, agriculture and health: a call for papers

**DOI:** 10.2471/BLT.22.288219

**Published:** 2022-04-01

**Authors:** Viroj Tangcharoensathien, Naoko Yamamoto, Rapeepong Suphanchaimat, Hathaichanok Sukbut, Somtanuek Chotchoungchatchai

**Affiliations:** a International Health Policy Programme, Ministry of Public Health, Tivanond Road, Nonthaburi, Thailand 11000.; b Healthier Populations Division, World Health Organization, Geneva, Switzerland.

Climate change is one of humanity’s most serious threats, putting at risk the functioning of the natural systems that sustain human health.^1^^,^^2^ In the Anthropocene, human activities have significantly altered the Earth through global warming, habitat loss and changes to the atmosphere. Based on a moderate emissions scenario that reflects little change from today’s development patterns, the average global temperatures will rise by 2.1–3.5 °C from preindustrial levels, which is above the 1.5–2 °C threshold set by the 2015 Paris Agreement.^3^ Although many countries committed to reduce carbon emissions and waste at the 26th United Nations Climate Change Conference and still aim at net-zero emissions, these commitments are insufficient to reach the target of keeping global warming within 1.5 °C above preindustrial levels.^4^ Despite scientific evidence, the gap between what we know and what we do in practice and political inaction continue to prevail.

The co-occurrence and synergistic interaction of climate change, loss of biodiversity and effects on food production have an exponential multiplier effect on human health compared to when these conditions are experienced separately. For example, food production and processing, retail, distribution and consumption, as well as food waste,^5^ contribute to climate change through the emissions of greenhouse gas. In turn, climate change affects food production and diversity and increases food insecurity, leading to overnutrition, undernutrition and deficiencies in micronutrients, particularly among children and vulnerable groups.^6^ Exposure to air pollutants increases all-cause, cardiovascular and respiratory morbidity and mortality, including lung cancer;^7^ effects are more significant among vulnerable subgroups.^8^

Changes in temperature and precipitation, and more frequent natural disasters are major factors of increased biodiversity loss.^9^ Meat production, intensive land use for agriculture and climate change also explain the decline in biodiversity.^10^ Intensive land use exposes humans to novel pathogens in wildlife^11^ and climate change alters host–vector–pathogen interactions, all of which increase risks of zoonotic diseases.^12^ While many health researchers focus on the implications of climate change on health and socioeconomic inequities, there remains much to understand about the repercussion of climate change on biodiversity, food production and human health.

The *Bulletin of the World Health Organization* calls for papers that address two broad and interlinked areas. First, the impact of climate change on biodiversity, food and nutritional security and human health. Second, effective policies and promising interventions that prevent, mitigate and provide alternative food production systems to minimize the health effects of these interlinked determinants. Analysis of the root causes of inaction in policies against climate change and its effects can contribute to effective policy levers.

The *Bulletin* welcomes contributions from all stakeholders, public health decision-makers, researchers, and civil society and community representatives, in particular those from areas most affected by climate change such as small island developing states and low-income countries. We welcome all types of papers that address the synergistic effects of climate change, loss of biodiversity and food insecurity on human health as well as its effect on different population groups or settings. We encourage manuscripts that identify effective and feasible policy interventions and national and global governance for health that address these determinants of health. 

The deadline for submissions is 1 July 2022. Manuscripts should be submitted in accordance with the *Bulletin*’s guidelines for contributors (available at: https://www.who.int/publications/journals/bulletin/contributors/guidelines-for-contributors) and the covering letter should mention this call for papers. This theme issue will be launched at the Prince Mahidol Award Conference in February 2023. 
